# Using focus groups with dairy cattle veterinarians to explore learning about calf welfare

**DOI:** 10.1017/awf.2022.7

**Published:** 2023-01-27

**Authors:** Christine L Sumner, Naseeb Bolduc, Marina AG von Keyserlingk

**Affiliations:** 1 Animal Welfare Program, Faculty of Land and Food Systems, The University of British Columbia, 2357 Main Mall Vancouver, BC, Canada, V6T 1Z6; 2 RNZSPCA, 199 Lincoln Road, Henderson, Auckland, New Zealand, 0610; 3 British Columbia Centre on Substance Use, 1045 Howe St Suite 400, Vancouver, BC V6Z 2A9, Canada

**Keywords:** animal ethics, animal welfare, discussion, group discussion, pedagogy, qualitative research

## Abstract

Dairy calf welfare is a growing interest within the veterinary field. However, a limited understanding of the conception of calf welfare by dairy cattle veterinarians can hinder efforts to promote welfare improvements on farms. The aim of this study was to explore how focus groups can promote learning about dairy calf welfare issues among cattle veterinarians. Focus groups (n = 5), that collectively had 33 participants representing five Canadian provinces and different geographical regions, were conducted as part of a continuing education workshop for Canadian cattle veterinarians. Two trained individuals undertook exploratory data analyses using applied thematic analysis and adult learning theory to develop a codebook of the data and identify the main themes. There were three main themes about learning that emerged from guided peer-discussion: (i) defining a shared concept of animal welfare from the veterinary perspective to diagnose the problem; (ii) understanding the problems of calf welfare by self-examination and group reflection; and (iii) negotiating the best approach to address the problems through sharing of ideas on improving calf welfare, including strategies for addressing welfare problems. In conclusion, focus groups can facilitate animal welfare learning within the veterinary profession.

## Introduction

Focus groups are a data collection method commonly used to gain a deeper understanding about a phenomenon under study. Focus groups are a relatively short, guided discussion amongst a small group of people and have become popular across a range of disciplines and towards exploring research questions (Wibeck *et al.* 2007). A number of studies have used focus groups to better understand perspectives on dairy cattle welfare, including dairy cattle professionals’ perspectives on animal welfare (Ventura *et al.* 2015, 2016), veterinarians’ professional obligations towards dairy calf welfare (Sumner & von Keyserlingk 2018), and dairy farmers’ views on calf welfare (Wilson *et al.* 2021). The use of focus groups in these studies aligns with how they are commonly used as a research tool, where the content of the discussion is the focus of analysis (Morgan 2010). Increasingly, the interactions of focus group participants are of interest to researchers, as analysing content alone overlooks the stand-out quality of social interactions and group-generated knowledge which produce the content (Wibeck *et al.* 2007; Farnsworth & Boon 2010; Kristiansen & Grønkjær 2018).

Under this shift towards analysing interaction (in addition to content), focus groups have been compared to group-generated, knowledge-based pedagogies such as problem-based learning to better understand learning processes (Wibeck *et al.* 2007). The pedagogical use of focus groups emphasises the participants ‘collective sense-making’ about a phenomenon (Wibeck *et al.* 2007; p 252). Focus groups use “collective engagement to promote dialogue and to achieve higher levels of understanding of issues critical to the development of the groups’ interests and/or transformation of conditions of existence” (Kamberelis & Dimitriadis 2011).

Interaction and discussion play an important role in numerous theories of adult learning. Knowledge is situated and exists in interactions among people (for a discussion, see Muro & Jeffrey 2008). When people are asked to participate in discussion, they bring with them their life experiences, which are inseparable from the discussion itself (Farnsworth & Boon 2010) and can be integral to the learning process. Transformative learning theory indicates that adult learners formulate beliefs based on experience, consider the context which characterise these experiences, seek consensus on the meaning of these beliefs, make decisions based on these beliefs, and become aware of one’s own and other’s biases that frame how one interprets beliefs and actions (Mezirow 2000). Additionally, transformative learning promotes dialogue by seeking to understand another’s point of view is part of the process of learning (Mezirow 2000).

A shift towards increased teaching of animal welfare in veterinary schools is occurring globally (Hernandez *et al.* 2018; De Briyne *et al.* 2020). However, there is need for more robust training around complex and multifaceted topics such as animal welfare ethics (Hernandez *et al.* 2018; De Briyne *et al.* 2020). Additionally, there is a need for ongoing training in application of concepts of animal welfare ethics and practical application of animal welfare science in clinical settings and on farms (Hayes *et al.* 2018; Hernandez *et al.* 2018). As discussed in Sumner and von Keyserlingk (2018), we found discussions with Canadian veterinarians indicated they acknowledged the need for more engagement about calf welfare with clients and in the clinic environment. In this previous paper we sought to understand the views of veterinarians; however, in the process of collecting data, it became evident that during the discussions, veterinarians were also developing their thinking about the nature of calf welfare problems, why they exist and persist, and what they as farmer advisors could and should do to address these problems. In Sumner and von Keyserlingk (2018), we did not report on how these discussions promoted dialogue and the collective sense-making about the calf welfare problems on farms. The purpose of this study was to explore how focus groups promote learning among veterinarians on the topic of calf welfare. Therefore, understanding how a guided discussion could drive learning on this topic can provide insight into using the method to further engage veterinarians in addressing calf welfare.

## Materials and methods

### Ethical approval

This study was approved by The University of British Columbia Behavioural Research Ethics Board under: #H16-00421. All participants provided written consent prior to participation.

### Researcher reflexivity statement

The authors of this study all have an interest in animal welfare and are motivated to improve it. At the time of data collection, CLS was a PhD student in the UBC Animal Welfare Program studying farmer and veterinarian motivation to improve dairy calf management and currently works as an animal welfare advocate and is located in New Zealand. NB was an undergraduate student studying philosophy at UBC and worked as a volunteer in the UBC Animal Welfare Program; and is currently a research co-ordinator at the British Columbia Centre on Substance Use, and MAGvK is a Professor of Animal Welfare located in the UBC Animal Welfare Program, with extensive dairy cattle welfare expertise. The mission of the Animal Welfare Program is to improve the lives of animals through research, education, and outreach. The first and third authors have previously published on the topic of veterinarian perspectives on dairy cattle welfare, which led to the current study of how methods such as focus groups can drive learning on this topic among this group of professionals. The focus groups were held at a continuing education workshop for veterinarians on the topic of dairy cattle welfare, where the first and third authors delivered the contents of the workshop, and the first author facilitated a focus group (workshop hosts also facilitated the remaining focus groups). Other topics in dairy cattle welfare were discussed in the workshop including lameness, access to pasture, and transition cow management, however, calf welfare was the only topic covered in the focus group discussion. Participants were aware of the author’s background and informed the goal of the research would contribute to a PhD.

### Study design

As stated above, this study builds upon the work first summarised by Sumner and von Keyserlingk (2018) that used focus groups to explore dairy cattle veterinarians’ views on calf welfare. The current research aims to explore how the group construction of those views is indicative of learning based on adult learning theories (further discussed below). The implications of using focus groups to promote learning among other stakeholder groups on topics such as animal welfare.

Focus group moderators facilitated the discussion using guided questions to help answer the original research question about veterinarians’ perspective on improving calf welfare reported in Sumner and von Keyserlingk (2018). The discussion guide included an introduction to the goal of the focus group, guidelines for providing feedback to the questions and other participants, and a review of the consent process. Probing questions (i.e., questions seeking clarification or further explanation) were included in the guide to prompt discussion on topics (i.e., social housing or bull calf management) not initially brought up by the participants. In addition to the themes reported in that study, the authors identified a number of instances that indicated the process of engaging in discussion about calf welfare promoted learning about the topic as distinct from analysing the content for the original research question. These learning-based phenomena were not reported in Sumner and von Keyserlingk (2018) and are the focus of the current study. Other studies have used a secondary analysis to take a second look at qualitative data sets when important phenomena emerge from data analysis that do not answer original research questions (Mills *et al.* 2021). We were inspired by Kristiansen and Grønkjær (2018) who, under similar circumstances, applied a different analytic lens to a focus group’s data set when the group interactions suggested were realised as an important function in the content that was produced during the discussion. We undertook this study to answer the following question: how do focus groups with dairy cattle veterinarians promote learning about calf welfare?

### Study site and participants

We conducted a 1-h focus group session with a convenience sample of 33 participants (five women, 28 men) during a continuing education workshop (facilitated by CLS and MAGvK) for Canadian cattle veterinarians on the topic of dairy cattle welfare. Participants represented five Canadian provinces from different geographical regions (Atlantic provinces, eastern provinces, and western provinces). The largest proportion of participants came from Ontario and Quebec, the two provinces with the largest number of dairy farms in Canada. Workshop attendees were asked in person to participate the day before the session was held and were offered to join a discussion group not audiotaped if they preferred to not participate. This ensured workshop attendees could still benefit from the discussion on calf welfare without joining the research aspect. All participants approached opted to join a research audio-recorded session.

We created five focus groups: one group of ten participants (French language group), and four groups of six participants (English language groups). One participant was not a veterinarian and their contributions to the discussion were omitted from the analysis. The French-speaking group provided bilingual participants with the option to discuss in their preferred language. All other participants were assigned to groups balanced for province to reduce homogeneity. To avoid biasing the participant views on calf welfare with those of the workshop hosts, the focus group session was the first time that calves were discussed during the workshop.

### Data collection and analysis

All focus groups were audiotaped and transcribed *verbatim.* The French audio file was first transcribed in French and then translated to English. All transcripts were checked against the raw audio files for fidelity and all discrepancies were addressed by the first and second authors and English- and French-speaking research assistants.

Our first step in our coding process was to use concepts of learning theory to provide an analytic model that would help us identify evidence of learning in our transcripts. We adapted Miettinen’s (2000) model of Dewey’s theory of reflective thought and action, which guided the identification of overall emergent themes. Dewey’s model of reflective thought and action describes cyclical processes that begins with the following steps: (i) noticing uncertainty; followed by (ii) defining the problem; then (iii) describing the conditions for how the problem has arisen or persists; followed by (iv) reasoning the problem and solutions to addressing the problem; and then finally (v) testing these solutions (Miettinen 2000). These steps occur in a cyclical process whereby once discussion has reached the point that solutions to identified problems are tested, the process of reflective thought and action begins again.

We decided to use Dewey’s model of learning to inform our analytical model because an initial review of the transcripts identified a series of examples that followed a similar pattern in the focus group data. Our emergent themes aligned with the steps noted above and this helped us conceptualise the cyclical nature of the discussion of calf welfare during the focus groups.

**Figure 1.**
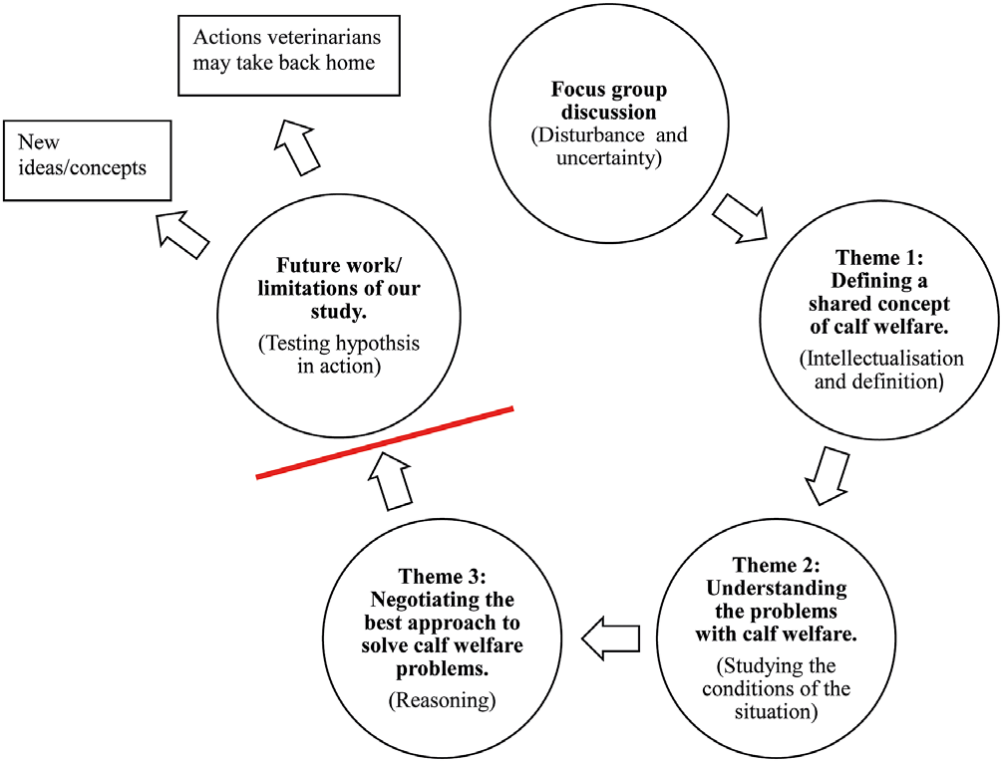
Thematic concept map for how focus groups promote learning among veterinarians on the topic of calf welfare. The current study did not include testing the hypothesis in action and is acknowledged as a limitation.

Following Guest *et al.* (2014), we coded the transcripts with the objective of identifying emergent themes based on the research question and informed by Dewey’s model of reflective thought and action as described Miettinen (2000). We also used the learning theories described by Mezirow (2000) and Kolb (1984) to inform more detailed coding of the data. For example, Mezirow (2000) emphasises processes of acknowledging one’s own frame of reference and other’s frames of references as integral to adult learning. Kolb (1984) emphasises the interplay of abstract thinking and concrete application of concepts as integral to adult learning processes. We used applied thematic analysis following a four-step process. We first: (i) identified emergent themes (descriptions of meaning in the text) in the transcripts; (ii) the first two authors created a codebook based on themes and an initial list of related codes (specific patterns of text that support the theme); (iii) coded all transcripts in detail using the codebook in Microsoft® Excel (2016) and updating it as needed; and finally, (iv) discussed and resolved any discrepancies in the coding. This was an iterative process where we open-coded the transcripts and then reviewed our coding process and outcomes through a theoretical lens with the steps in Dewey’s model of reflective thought and action. Figure 1 is our thematic map presented in a cyclical process that aligns with Dewey’s model.

After the first codebook was created, the first two authors adapted Dewey’s model of reflective thought and action to refine the emerging codebook and to capture the cyclical patterns of learning emerging in the data analysis, including several stages of learning where problems are defined, understood, and addressed (see Miettinen 2000). Our final codebook reflects that each major theme is a variation of processes in Dewey’s model of reflective thought and action. We assumed that the act of participating in the focus group discussion would correspond to Step 1 in Dewey’s model, where there is acknowledged disturbance and uncertainty about a phenomenon. The point of the focus group was to discuss dairy calf welfare, a topic often overlooked by the veterinary profession and, in practice, on farms (Sumner *et al.* 2018). Asking them to discuss the topic was an act of disturbing their normal, habitual practice of not typically engaging with each other on this topic. However, as the transcripts are the only source of data, we recognise that our thematic model is limited, as we were not able to include a step where participants are testing their hypothesis in action (Step 5 in Dewey’s model); this latter step was beyond the scope of the focus group discussion. This discrepancy is indicated with the dashed red line in our thematic model. Figure 1 presents of thematic conceptual map for this study.

Saturation, where no new information emerged, was achieved during data collection and analysis (see Saunders *et al.* 2018). During data collection, clarification was sought by group moderators to encourage elaboration of an utterance, participants were asked for further comment before moving on to ensuing questions in the guided discussion, and a final invitation to add any additional comments was offered at the end of each group’s discussion. Saturation of themes was achieved through an iterative process between authors that coded the data, and until no new themes or sub-themes emerged from the analysis. Exemplars illustrating the sub-themes for each major theme are presented in the following section. Quoted passages have been modified to remove redundant speech or non-starters and are indicated with ellipses. Brackets are used to indicate where authors have clarified speech. The participant identifiers and group numbers are included in the results but have been further randomised from the data collection session.

## Results

We identified three primary themes related to learning that emerged from the guided peer-discussions: First, participants sought to define a shared concept of animal welfare from their own experience; second, participants further sought to understand the nature of calf welfare problems; and third, participants negotiated the best approach to solve identified calf welfare problems.

### Theme 1: Defining a shared concept of calf welfare for the veterinary perspective

During the first step in learning about calf welfare issues on farms, participants engaged in a process of defining a shared concept of calf welfare in general and current welfare problems. This was an iterative process among participants where the meaning of a welfare problem was created collectively and then tested against their perceptions of other groups’ (e.g. farmers, public) understandings of welfare.

### Collectively creating meaning

In response to the question of what mattered for calf welfare, respondents offered suggestions they considered were features of calf welfare or a possible definition of welfare and, often, other participants responded in agreement or disagreement. These suggestions were offered as examples of practices, affective states such as hunger, personal experiences related to practices, such as performing a painful procedure, their own observations of the animals and the farm environment, and through use of animal welfare frameworks. At times, respondents offered suggestions, or responded to suggestions by identifying a change in views reflecting evolving concerns about calf welfare.

The passage below includes interactions between participants in Group 5 where they discussed hunger as a welfare problem for calves and how the use of physical and behavioural observations informs a welfare concern. The participating veterinarians offer their experiences on farms or in places where calves are sold through sales barns and often transported from their place of birth within the first few weeks of life and thus still dependent on milk. Defining hunger as a welfare issue is first suggested as an inadequate source of nutrition by Vet 3. The conversation then includes suggestions of the calf’s body condition and behaviour to support defining hunger, as related to inadequate milk allowance, as a welfare problem. Finally, the participants further discuss the relative harm of hunger and conclude by coming to agreement that it is indeed a welfare problem.

Vet 3: …*As far as nutrition, …I have problems with that, a big problem with that, personally. … They drink two litres of [milk] — it’s gone, and then the rest of the day they have nothing, it’s not right.*

Vet 6: *If you go into those barns and those calves are there, I know they’re conditioned to be fed when someone comes in the barn, and so they’re going to be bawling and active and looking for something, but when they’re not fed enough, that’s pretty intense.*

Vet 3: *And they’re thin.*

Vet 6: *Those calves are really… looking for something because they’re hungry. Not just because somebody came in the barn and they get fed when somebody comes in the barn, but they actually are hungry. … Those calves fed twice a day, two litres, that seems to be — is it — is it a pain? …Is that a welfare issue for these calves? Probably a bit. There [are] worse things, right? A lot worse things.*

Vet 3: *Sure, but hunger’s not a great —*

Vet 6: *Hunger’s what?*

Vet 3: *I think it’s a welfare issue.*

Vet 6: *Well, yeah.*

Vet 3: …*I think if they’re getting four litres a day, they are hungry.*

Vet 6: *Yeah, I agree with you…. But even the better-fed calves will exhibit hunger behaviours when you come into the barn in the middle of the day.*

Vet 3: *But if you’re in there for an hour, dehorning, like, you see them sucking, the whole time.*

Vet 6: *Yeah, yeah. Oh, yeah, they’re wanting something for sure. …We also do sales barn inspections, and you’ve got to check all those bob calves for the sale, so we check for attitude, navels, take temperatures, listen to lungs if we need to. And it’s terrible. You go into these pens and these calves, a lot of them, …you know which ones haven’t been fed that morning or don’t get enough. They just mob you. They won’t leave you alone. …and other calves are just lying there, content, because they had a good meal that morning and they’re fine.*

### Identifying alternative meanings

Defining calf welfare problems also included discussing what others would consider important to welfare, for instance, many of the focus groups referred to what calf welfare means to the farmers or the public. This process allowed the group to test the boundaries of their perspectives on calf welfare, contrasting their views with those views they ascribed to farmers and the public, but also at times aligning with the views. Participants’ ability to identify and consider alternative meanings of welfare, and either contrast or align with these different perspectives, further helped define their own group understanding of calf welfare. In establishing boundaries as veterinarians and professionals, participants also explored and tested their group values and norms as related to calf welfare problems.

In the passage below, three participants in Group 2 discussed perceptions of farm hygiene while identifying this issue as a welfare problem for calves. In addition to their own perspectives as veterinarians, their identification of this as a problem included how they felt the public and farmer would view the issue. This conversation below picks up after the group had started discussing the use of automated calf milk feeders and how they have benefited the drive for improvements in farm management (and arguably welfare) but that increasing milk allowance comes with a challenge of maintaining pen hygiene resulting in welfare issues for calves:

Vet 1: *Well, … [with automated calf milk feeders] you have to clean the pens more often because they’re [the calves] drinking more [and thus urinating more], so there are wetter pens, so you got to take care of that as well, right?*

Vet 3: *I think [about] the welfare part to it too, right? …I have places where I wouldn’t have a problem taking someone from the general public that has no idea about how calves are raised, and I have a big chunk of farms I would never want anyone to go in to see, like, [how] these cute, little, baby calves are being raised like, right? … And it’s hard because even — some of the farmers understand it and they’re working on building new housing, but a lot of them, I don’t know, …because they see it every day, they don’t think it’s that bad? I don’t know. …Or they’re just blind to it, right? They just think that’s the way it’s always been and there’s nothing different.*

Vet 4: *Some of those guys are the hardest to change, I find.*

Once participants had defined a welfare problem, the next step was to better understand the conditions for why the problem existed. This is discussed below in Theme 2.

### Theme 2: Understanding the problems with calf welfare

During the second step of the learning process, participants engaged in self-examination about their values and practices related to animal welfare. After engaging in discussion that led to defining the welfare problem, this step involved attempting to understand the conditions of the problem more deeply by reflecting on both one’s own beliefs and practices, and those of other members of the group.

### Personal reflection

In their effort to understand the conditions of problems with calf welfare, participants often questioned their own frames of reference such as how their background, such as their education or culture, influenced how they think about and work on welfare. Notably, these frames of reference were portrayed as both positive and negative influences in relation to calf welfare. In addition to these frames of reference, participants offered reasons for their beliefs about calf welfare. This included reflection on the systemic causes of the problems, and other social influences such as gendered norms. Participants also specified the practical challenges they face in their day-to-day lives that make it difficult to address calf welfare in practice.

In the passage below, we see two participants in Group 3 personally reflect on changes in veterinarian attitudes towards mitigating pain during disbudding. Calves are routinely disbudded on farms and, in many parts of Canada, this is performed by the farmer. The practice is painful for the calf, and pain mitigation such as anaesthesia and analgesia may be used, however, it is not required under law. This passage demonstrates all three aspects of the personal reflection: questioning their frame of reference (their veterinary education); offering reasons for their beliefs about welfare, such as the need for pain management; and identifying practical challenges in addressing welfare such as perceived resistance from their clients with increasing the cost of a visit. The participants also discuss feelings of guilt, and attempts to assuage these feelings while reflecting on the evolving considerations for pain from disbudding as a calf welfare issue:

Vet 1: *There’s some youngster producers [farmers] coming in the market and they are really open to the idea of pain management… the younger vets are pushing [for the use of pain relief], …and if I don’t do the job well or I do the same thing I did the last twenty years, they … comment.*

Vet 5: *I think you’re right as well. We’ve had a bunch of new vets come in, it’s part of their training, they just can’t believe that we haven’t been doing pain management …so then you start to feel a little guilty, right? It’s like, okay, …I mean, I value the education that we’ve received at school. I think it’s been top notch, …anyway, there’s just more weight put on pain management, and I think it’s valid. …I guess I’m in the mindset that I want to see the benefit of it to the producer as well as to the calf. If they’re showing me that this calf is going to do better, and if the calf’s going to do better, then I know it’s going to be profitable for the producer, then I’m on board. …I’m slowly — I’m changing.*

Vet 1: … *You [used] this word, ‘guilty’ a few minutes ago, and I feel the same a little bit. But the profession has evolved too because if I go back twenty years ago, my idea was to get [on] the farm because not all the producers [using] preventive medicine … I think youngster veterinarians are good at pushing us to do better pain or welfare stuff, but we did the first part of the job [which] is getting [on] the farm and selling the idea of preventive medicine. Now it’s easier for them… to talk pain management. It’s an opportunity for us to change, but I’m not sure it will be easy…*

Vet 5: *Twenty years ago?*

Vet 1: *—Twenty years ago. I’m not sure it would have worked because it’s still tricky … to change…for me, it’s almost a new concept.*

Vet 3: *Yeah, I agree.*

Vet 5: *Yeah, I agree.*

Vet 1: *…I’m just trying to feel less guilty.*

### Group reflection

Group reflection on the problems of calf welfare occurred when participants would respond to others by probing for reasons or seeking more information to understand the conditions of the problem. Often, this would lead to participants seeking agreement or validation from others when describing the conditions of their work and the frames of reference for their beliefs about animal welfare. Through group reflection, participants also assessed the differing contexts within which they think about and work towards welfare. Between group members, participants noted differing geographical and cultural contexts that influenced their beliefs and responses to problems with calf welfare.

In the following discussion, participants in Group 2 reflect on the conditions of bull calf welfare and the underlying reasons for why they experience poor welfare with mention of routine practice of transporting them from the location of their birth at an early age to a rearing facility or sales barn. Here, we see the participants discussing how the value of the bull calf as a product will drive the quality of care provided at a young age:

Vet 6: *There’s a lot of guys that feel that if they know they’re not keeping the bull calves and they don’t know where they’re going, then, soon as that navel’s dry or the calf’s dry, it’s on the truck and gone, right?*

Vet 2: *They might not even really get colostrum.*

Vet 1: *They’re not getting colostrum sometimes.*

Vet 3: *It’s price dependent. [indiscernible] three or four hundred dollars, they get lots of attention. They get, you know, [antibiotics], lots of colostrum. If they’re worth twenty bucks, they’re — you know, they get fed, sort of.*

Vet 5: *Yeah, we found with calf prices last year, when some guys were getting six hundred bucks for a veal calf, … they were paying attention.*

After collectively defining a welfare problem, and reflecting on the conditions of the problem, participants would begin to discuss and negotiate the best approaches to solve the problem. This is described in Theme 3.

### Theme 3: Negotiating the best approach to solve calf welfare problems

During the final step in learning that we observed, participants engaged in a process of negotiating the best approach to solve the specific calf welfare problem under discussion. After defining the problem, and attempting to understand the conditions of the problem, this next step involved participants collectively developing solutions for addressing the problem. This process included participants both engaging in processes of offering abstract concepts as solutions, as well as concrete examples from their own experiences as veterinarians.

### Abstracting solutions

Participants offered solutions to addressing calf welfare problems that were ideas not yet implemented, rather as practices suggested as possible ways to improve the condition. At times, participants sought agreement from the group, and these were met with confirmation or disconfirmation from other respondents as to whether these ideas would work (or not). In these exchanges about abstract ideas, participants also asked for advice, which frequently bridged to more concrete suggestions.

In the passage below, we see the members of Group 1 working together to find a solution to their identified problem of calves being too young when they are transported from the farm to the sale barns. The participants theorised that introducing a minimum age or bodyweight for calves before shipping them to the sales barn would create an incentive for farmers to wait until the calves are older (and less vulnerable) and feed them more food before selling them.

Vet 1: *The more I think about it, I like the idea of somebody not being able to sell their calves until they’re two weeks of age.*

Vet 5: *I like that one too.*

Vet 3: *Or three weeks.*

Vet 5: *Three weeks would be even better.*

Vet 1: *So yeah, so I think that would solve a lot of issues, right?*

Vet 3: *Yeah, from the bull calf side of things, it would.*

Vet 5: *Probably you almost would need in these circumstances a weight. Because you know, like, any one of us here, they’ll try to cheat, and [say], ‘It was born, you know, two weeks ago.’ Well, come on. So, you go maybe by weight or their minimum weight.*

Vet 1: *Oh, yeah, yeah, yeah.*

Vet 5: *Say, okay, you need, let’s say, a 150 lbs. Oh, boy. There are some people that will keep those calves for a while, and they say, ‘Well, how can I manage to get that weight faster?’ We’ll say, ‘Give more milk,’ right?*

Vet 1: *Yeah, yeah, yeah. No, that’s a good way to look at it.*

Vet 3: *I like that. I like that.*

Vet 5: *And give more milk but give better nutrition. It’s not only more milk, it’s the whole picture, right?*

Vet 1: *Yeah. Else the calf won’t reach that point, right? A poorly managed calf won’t reach that.*

Vet 5: *Exactly. They won’t.*

Vet 3: *Not quick, anyways.*

Vet 5: *Not quick.*

Vet 1: …*So I think that actually that would be a good idea.*

Vet 5: *Uh-huh. … That would solve a lot of problems.*

Vet 1: *Yeah, yeah. No, do it by weight.*

### Concretising solutions

Suggestions of current strategies usually consisted of concrete examples of practices that participants had already implemented and, in ways, tested as solutions. Concrete solutions were also offered as methods to facilitate certain practices, such as communication attempts or a demonstration of a concept. Participants also shared their experience as social influencers, that their demonstration of caring about a problem was an important part of engaging with their clients to care about a problem.

Participants in Group 4 discussed how they sedate their calves with Rompun and use lidocaine as a local anaesthetic during disbudding procedure to address the problems of pain and stress due to handling. They also described how they teach their clients to provide local anaesthetic and sedate their own calves as a way to promote pain relief for the calf.

Vet 6: *We’ve recently started dispensing diluted Rompun. I don’t know how many of you [do that].*

Vet 3: *We do that.*

Vet 4: *We mix Rompun with our lidocaine, and so when the calf gets its [injection for nerve] block, it also is getting sedated. … and they wake up and wonder what happened, right? Sort of. They have an idea. They’re not that sedated.*

Vet 3: *Yeah, I do think the sedation does help probably, as you said, like, there’s less of a negative association with the person who did the unpleasant procedure [disbudding], right, if they’re sedated rather than just doing a [local anaesthetic]. So, we also use a lidocaine-Rompun mixture … and yeah, I think the calves probably accept that very well, and recover.*

Vet 2: *It’s so much easier to do. You’re not struggling with the calves, plus, it’s easier on the operator, and usually on the calves in the long run.*

Vet 3: *Yes. Yeah.*

The process of negotiating solutions to calf welfare problems was demonstrated through an iterative process of discussing abstract and concrete actions. Respondents were able to share their experiences with others and think through potential solutions in discussion with their colleagues who could offer their own lived experiences with solutions.

## Discussion

We conducted a secondary data analysis on focus group session using different adult learning theories to explore how focus groups can promote learning among veterinarians on the topic of dairy calf welfare. We identified key aspects of learning theory that reflect the strength of focus groups to promote learning, including using one’s own language, concepts, and concerns to discuss a topic, deeper elucidation of the topic, and a collective sense-making about the topic (see Wilkinson 1998; Wibeck *et al.* 2007). We also observed how focus groups inspire change in addressing a perceived problem (see Kamberelis & Dimitriadis 2011).

### Defining a shared concept of animal welfare for the veterinary perspective pushes the boundaries for veterinarians’ perceptions of a problem

In defining a shared concept of animal welfare, specifically for dairy calves, we observed the participants learning to expand their assumed focus of concern for calves, which typically focused on biological functioning, to also include constructs such as affective states, and naturalness (Fraser *et al.* 1997). In doing so, the group brought out tacit assumptions of what they considered as aspects of calf welfare (see Mezirow 2000) and tested new ideas in a format that allowed group member feedback. Discussing what matters for calf welfare in this context allowed participants to engage in problem definition, where tentative understandings of a phenomenon are revealed (Miettinen 2000). This step is needed before formulations about how to address a problem can be identified.

In previous work undertaken by our group, Ventura *et al.* (2016) and Sumner and von Keyserlingk (2018) found that veterinarians are concerned about a shared concept of welfare. In the current study, the discussion also revealed group values heavily centred on health and many interpretations about calf welfare are based on a concept of physical health. For cattle veterinarians, this seems reasonable due to the emphasis on physical health as fundamental to their education, daily duties, and role in the food industry (Sumner *et al.* 2018). In Sumner and von Keyserlingk (2018), veterinarians identified affective states, naturalness, and socialisation with their mother and other calves to be welfare concerns for dairy calves. In the current study, we found the process of discussion with their peers allowed participants to engage in learning processes that helped them to further define calf welfare to include affective states such as pain and hunger. Most interesting was that participants sought boundaries for defining these concepts of calf welfare; frequently relying on the perceived views of other groups such as the public, farmers, and industry members. These other stakeholders served as either points of divergence or convergence on the topic. During this process, the affirming/disaffirming of suggestions, and contrasting/aligning with other groups also suggests dialogue within the group of participants, and that there was room for disagreement. A challenge with focus groups is to avoid reaching consensus too easily and avoid ‘scaffolding of group think’; or ‘premature closure’ (Kamberelis & Dimitriadis 2011). One concern of using focus groups as a pedagogical tool is the potential for conversations to be dominated by a few (Stewart *et al.* 2007), and the perpetuation of misinformation. Although this is not a universal concern for all topics (eg the sharing of personal experiences as part of a discussion), care should be taken in the moderation of the focus group so that factual information is scrutinised to avoid perpetuating the use of outdated or incorrect information.

The focus group format also provided the veterinarians with the opportunity to explore other shared concepts of calf welfare, with implications of these serving as social norms. Social norms are beliefs about behaviour contingent on group values and reinforced with either positive or negative sanctions (Southwood & Eriksson 2011; Bicchieri 2017). An issue remains as to how norms and normativity are understood as constructions of a focus groups discussion, and this is in part due to methodological limitations associated with the analysis, but also epistemological orientation towards focus groups (Kristiansen & Grønkjær 2018). To explore learning on the topic of calf welfare, we included analysis of the interactions between participants, but did not use an explicit analytic approach such as conversation or discourse analysis. We suggest our approach identified where participants’ discussions of calf welfare revealed elements of social norms, but further exploration is needed to better understand these phenomena, and their role in improving calf welfare.

### Understanding the problems with calf welfare through an iterative process between the individual and the group

Theme two was strongly characterised by learning as a process of searching for common understanding and justification for interpretation (Mezirow 2000). By participating in focus groups, participants were able to work together to better understand calf welfare problems on dairy farms.

Learning includes joint elaboration leading to the co-construction of knowledge about a phenomenon (Wibeck *et al.* 2007). Participants share thinking processes in which they exchange information, modify views, and develop arguments leading to a shared understanding of the problem (Wibeck *et al.* 2007). In the current study, this step forward in learning allowed participants to formulate a deeper understanding of the conditions for why calf welfare issues exist on dairy farms, and also preceded the development of strategies for how to address these problems.

By asking participants to discuss calf welfare, we were in fact asking them to step outside their habit of thinking of calf welfare on farms, as part of their routine professional experience. The discussion itself was the starting point for challenging their frame of reference on calf welfare, informed by their experiences and interactions in their daily lives. When participants discussed how their background influenced their views on calf welfare, they in part were questioning their own frame of reference. By acknowledging our own point of view, we engage in processes that transform our ‘taken for granted’ frames of reference, leading to a deeper understanding of a phenomenon as it becomes more inclusive of others’ views and experiences (Mezirow 2000). In the current study, veterinarians often engaged in discussions that included seeing the topic from another’s point of view, typically farmers, but also the public and industry groups. This point is made with a degree of caution and the participants were unable to fully engage in transformative learning, because these focus groups were not designed to include members of the public or farmers. We make this point to note that even as a homogenous group, veterinarians engaged in processes of acknowledging that calf welfare concerns are likely dependant on the perspective of the stakeholder; in the current study they used the perspective of the public or farmers as a means to test their own assumptions.

Homogenous groups can reinforce their own frames of reference where there is a lack of constructive dialogue and inclusion of others’ views, where non-group members are present, or discussion is structured so that more robust dialogue is limited (see Mezirow 2000). Reinforcing current frames of reference can also occur if focus groups end up reinforcing current social dynamics in a group, lead to the dominant voices controlling the discussion, and potentially leading to participants not feeling comfortable sharing their views and experiences (Stewart *et al.* 2007). For this study, there were numerous instances where participants engaged in group reflection to explore a specific calf welfare topic as a means to better understand it. Future work pairing veterinarians and members of the public to further discuss calf welfare could promote more transformative learning on the topic where more inclusive views of calf welfare are realised and guide veterinarian actions.

### Negotiating the best approach for addressing calf welfare through conceptualised and the lived experience

Focus groups allow key problem-solving processes to occur which contribute to a collective sense-making: through discussion, participants activate prior knowledge and elaborate new knowledge about a topic (Wibeck *et al.* 2007). In our study, problem-solving emerged as the last part of this process and is described in Theme 3.

We observed activation of prior knowledge with participants frequently offering solutions to addressing calf welfare based on what they were already doing on their clients’ farms. We observed the elaboration of new knowledge with participants identifying how other participants’ approaches could work in their own practices, or further elaborated on other ideas tangential to those already discussed. This iterative process emerged as a key theme, however, the cycle of first defining the problem and better understanding the problem prompted the problem-solving stage. Participants had already defined many concerns as calf welfare problems, identified poor communication with clients as key reasons for why they exist, and thus by this point, were able to assimilate this new co-constructed knowledge into a problem-solving process which led to strategies of improved farmer engagement.

The process of abstracting and concretising ideas is described as part of the learning process where knowledge is constructed in part through an iterative relationship between conceptual and actual experience (Kolb 1984) and indicates the cyclical nature of reflection and application. Miettinen’s (2000) interpretation of Dewey describes this step, in learning, as a group testing their collective working hypothesis of how to address the problem. We note that our inclusion of how veterinarians address issues such as pain management through teaching farmers to use anaesthetics may not be universally accepted practice, however, this example can still serve as impetus for veterinarians to explore other ways to improve practices that impact calf welfare. The tone of discussion for Theme 3 had an emphasis on what to do when participants returned to their routine advisor role on dairy farms. As noted earlier, a limitation to understanding the full extent of learning, is not knowing what participants did when they returned to their normal routines as veterinarians. This is a limitation to our study with understanding how the reflection and thought manifested in action back home in their clinics, and on their clients’ dairy farms. Unfortunately, we do not know if participants changed their behaviours after the focus group session. This limitation can be addressed in future studies by following up with individuals or ongoing group discussions.

### Implications for future work

Our aim here was to explore how focus groups prompted learning among dairy cattle veterinarians on the topic of calf welfare. We found learning occurring through a process of first defining problems, then more deeply understanding these problems, and finally negotiating solutions to calf welfare problems. In the process of providing more in-depth accounts and socially constructing the meaning of phenomenon (Wilkinson 1998), we argue participants are changed by a focus group discussion; they have engaged in learning, and now better understand the phenomenon. Using focus groups to facilitate the social construction of meaning about a particular topic that is limited in understanding among stakeholders, such as veterinarian understanding of calf welfare, can support applied and translation sciences, and extension work, where a goal is to inspire change (see Kamberelis & Dimitriadis 2011). Future work could include assessment of learning through focus groups and application of learning post-discussion.

## Animal welfare implications and conclusion

Traditionally, focus groups are more commonly used to analyse the content generated in discussions, however, analysing groups interactions indicates they are a useful tool for other purposes, including facilitating learning. Veterinarians play an important role in stewarding animal welfare improvements and are key advisors to farmers. Our study indicates focus groups can be used to promote learning among veterinarians on topics of calf welfare, and thus has the potential to greatly impact farm animal welfare.
